# Triglyceride glucose-waist circumference: the optimum index to screen nonalcoholic fatty liver disease in non-obese adults

**DOI:** 10.1186/s12876-023-03007-8

**Published:** 2023-11-02

**Authors:** Shuying Li, Li Feng, Jie Ding, Weihong Zhou, Tangbin Yuan, Jiangfeng Mao

**Affiliations:** 1 Department of Health Management Center, Nanjing Drum Tower Hospital, Affiliated Hospital of Medical School, Nanjing University, No 53 Zhongshan North Road, Gulou District, Nanjing, 210000 China; 2 General Surgery, People’s Hospital of Ganyu District, 88 Haicheng Road, Qingkou Town, Ganyu District, Lianyungang, 222100 China; 3 Department of Endocrinology, Peking Union Medical College Hospital, Chinese Academy of Medical Sciences, Peking Union Medical College, No 1 Shuaifuyuan, Dongcheng District, Beijing, 100730 China

**Keywords:** NAFLD, Non-obese, TyG, TG, HDL-c, Control attenuation parameters

## Abstract

**Background:**

Non-alcoholic fatty liver disease (NAFLD) is easily neglected in the non-obese population. TyG index (triglyceride glucose Index) and TG/HDL-c (triglyceride to high-density lipoprotein cholesterol) are new indicators to evaluate insulin resistance (IR). Fibroscan is a non-invasive way to assess hepatic steatosis [by control attenuation parameters (CAP)] and fibrosis [by liver stiffness measurement (LSM)].The purpose of this study was to explore the correlation of TyG and its combination with obesity indicators [TyG-waist circumference (WC), TyG-body mass index (BMI)] and TG/HDL-c with CAP and LSM.

**Method:**

One thousand seven hundred seventy-six adults (age ≥ 20 years, BMI < 30 kg/m2) in the National Health and Nutrition Examination Survey (NHANES) 2017–2018 were included. The correlations of CAP and LSM to the indexes were assessed by generalized linear models.. Receiver operating characteristic (ROC) curve was performed to evaluate the diagnostic capability of the indicators on NAFLD and liver stiffness.

**Results:**

Survey-weighted percentage of NAFLD in non-obese was 38.6%. In the fully adjusted models, there were positive associations of TyG, TyG-BMI, TyG-WC and TG/HDL-c to CAP, with the βs of 24.810, 0.704, 0.29 and 2.983 (all *p* < 0.05), respectively. There were positive associations of TyG, TyG-BMI, TyG-WC, and TG/HDL-c to NAFLD, with ORs of 3.387, 1.03, 1.010 and 1.281 ((all *p* < 0.05)).The positive association was detected for TG/HDL-c and TyG-WC and LSM with βs of 0.057 and 0.004(*p* = 0.021 and *p* = 0.003).TyG-WC were positively associated with liver stiffness with OR of 1.006(95%CI = 1.002, 1.012). Furthermore, the TyG-WC had the strongest diagnostic capability (ROC = 0.806; 95%CI: 0.785–0.826) on NAFLD in non-obese participants, with a specificity of 0.737 and sensitivity of 0.746.

**Conclusion:**

In US non-obese population, the TyG, TyG-BMI, TyG-WC, and TG/HDL-c are positively correlated with CAP and NAFLD. TyG-WC has clinical importance in identifying NAFLD in the non-obese population.

**Supplementary Information:**

The online version contains supplementary material available at 10.1186/s12876-023-03007-8.

## Introduction

NAFLD, which is supposed to be one component of metabolic syndrome, affects approximately 25% of adults worldwide [1]. NAFLD will become the main cause of end-stage liver disease in the coming decades and exert great economic pressure on the global health care system [2, 3]. Therefore, early identification of NAFLD is of vital importance for the intervention. NAFLD usually goes like the shadow following obesity. However, it also insidiously occurs in non-obese individuals [4]. The occurrence of NAFLD in non-obese subjects is easy to be neglected. Globally, the prevalence of NAFLD is 3%—30% among non-obese subjects, defined as BMI < 25 kg/m^2^ in Asian and < 30 kg/m^2^ in the Occident [5]. Therefore, simple and convenient indicators are required to identify NAFLD in non-obese individuals.

TyG and its combination with other obesity indicators (TyG-BMI, TyG-WC) and TG/HDL-c are emerging indicators for evaluating insulin resistance (IR) in obese or type 2 diabetes mellitus (T2DM) individuals [6]. IR closely correlates with NAFLD, and the prevalence of NAFLD in T2DM is fivefold higher than those without diabetes [7]. However, in the non-obese population, the relationship between the above emerging indicators and NAFLD has not been investigated.

Due to the asymptomatic nature of NAFLD, early detection has proven to be challenging, particularly in non-obese individuals. Liver biopsy has traditionally been regarded as the definitive method for diagnosing NAFLD, but its invasiveness and high cost have hindered its widespread use [8]. Large-scale epidemiological studies mainly use liver enzymes and ultrasound to screen NAFLD. However, these indexes have low sensitivity and cannot identify high-risk NAFLD [9, 10]. Vibration-controlled transient elastography (VCTE) can quantify liver fat by using the controlled attenuation parameter (CAP), with a sensitivity of 87% and a specificity of 91% for detecting hepatic steatosis [11]. Liver steatosis (CAP) calculated by transient elastography (FibroScan) is an evidence-based noninvasive method to evaluate NAFLD [12]. Meanwhile, liver stiffness measurement (LSM) using VCTE has emerged as a commonly employed technique in gastroenterology and hepatology clinics [13]. While there are simple indicators available, such as the AST to-platelet ratio index (APRI) and Fibrosis-4 (FIB-4) index, to assess liver fibrosis, there remains a dearth of effective and straightforward indicators to identify liver steatosis specifically in non-obese populations [14].

This investigation was conducted to evaluate the prevalence of NAFLD using FibroScan in non-obese individuals. The relationships between TyG, TyG-BMI, TyG-WC, TG /HDL-c, and CAP were investigated. Furthermore, the diagnostic capabilities of the indexes on NAFLD and liver stiffness in the non-obese US population were also detected.

## Methods

### Data sources

The NHANES (National Health and Nutrition Examination Survey) is a population-based cross-sectional survey designed to collect information on the health and nutrition status of adults and children in the United States of America. Most of the data in NHANES is freely accessible to researchers worldwide. This investigation extracted data from 2017 to 2018.

### Study population

**Fig. 1:**
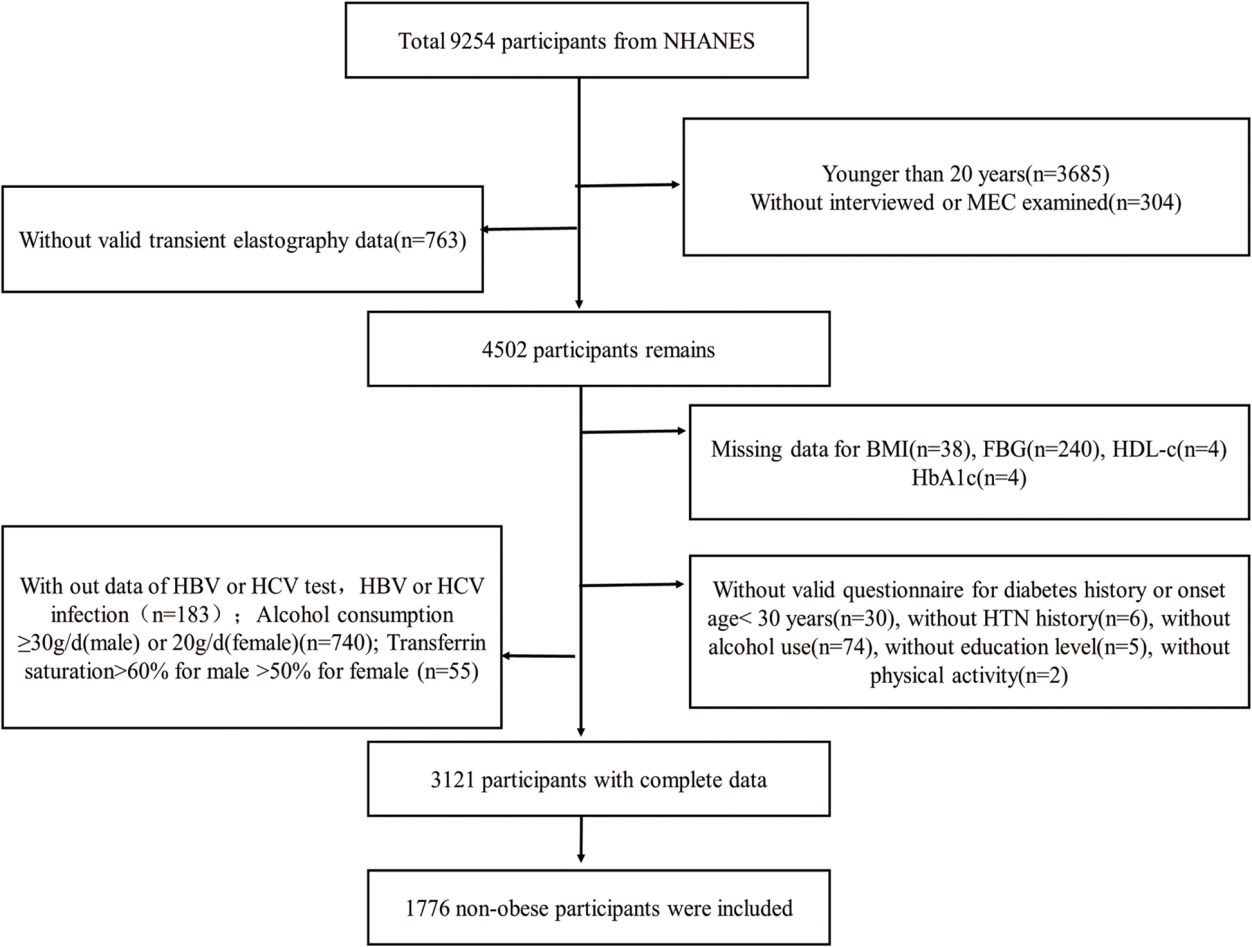
Flow chart for the population included in the study

A total of 9254 persons were enrolled in NHANES from 2017 to 2018. Among them, 3685 persons were excluded for younger than 20 years and 304 participants were excluded for incomplete survey data. Another 763 persons were excluded for lack of transient elastography data. A total of 403 individuals were excluded for lack of laboratory examination data (FBG, HDL-C, HbA1c), anthropometric data (BMI), invalid questionnaire data (history of diabetes, history of HTN, alcohol use, education level, physical activity), and individuals with diabetes onset age less than 30 years old (to minimize the confounding factor for type 1 diabetes mellitus). Individuals (*n* = 183) were excluded because of no data of HBV or HCV test and with HBV or HCV infection (positive serum hepatitis B surface antigen and positive serum hepatitis C antibody). Individuals with alcohol consumption ≥ 30 g/d (male) or 20 g/d (female) were also excluded (*n* = 740). Participants with transferrin saturation > 60% for male and > 50% for female were also excluded (*n* = 55). Finally, 1776 individuals (BMI < 30 kg/m^2^) were included for investigation. See details in Fig. 1.

### Study variables

The index for our investigation was TyG, TyG-BMI, TyG-WC, and TG/HDL-c. TyG was calculated by formula of Ln[TG (mg/dL) × FPG (mg/dL)/2]. TyG-BMI was calculated by TyG × BMI and TyG-WC was calculated by TyG × WC. TG/HDL-c was defined as the ratio of TG to HDL-c. T2DM was defined by self-report or by HbA1c ≥ 6.5%. Hypertension was defined by self-report. Physical activity status was defined as a continuously vigorous and intensive activity for at least 10 min in a typical week. Education level was stratified according to whether he or she had attended high school. BMI < 30 kg/m^2^ was defined as non-obese. CAP ≥ 248 dB/m was defined as NAFLD [15]. The examination was considered reliable when a minimum of 10 valid measurements were acquired, with an interquartile range/median LSM ratio of less than 30%, following a fasting period of at least 3 h. 24-Hour Food Recalls were conducted to get data on alcohol intake. Sociodemographic variables such as age, gender, and race were extracted from the file named demographic variables. BMI and WC were extracted from the file named body measures. FBG, HbA1c, Total cholesterol (TC), TG, HDL-c, Alanine aminotransferase (ALT), transferrin saturation, hepatitis B, and hepatitis C were extracted from the laboratory data. Alcohol intake was extracted from dietary data.

### Statistical analyses

Analysis was performed by R packages and Empower Stats (http://www.empowerstats.com). Weighted analysis was performed using survey weights of NHANES. The association of TyG, TyG-BMI, TyG-WC, and TG/HDL-c with NAFLD (reflected by CAP) and liver stiffness (reflected by LSM) was evaluated by the generalized linear regression model. The models were adjusted for age, gender, race, alcohol use, education level, physical activity, BMI, HTN, diabetes status, and TC. The diagnosis indicators and determine the threshold value were evaluated by ROC curve analysis. Analysis of Variance (ANOVA) was conducted for evaluating the prevalence of NAFLD in different quantiles of TyG-WC groups. *P*-value < 0.05 is defined as a significant difference.

## Results

Participants were divided into two groups according to NAFLD or not. The survey-weighted percentage for NAFLD was 38.6% (95%CI: 33.9%, 43.3%). The mean CAP was 291.7(287.7, 295.6) dB/m in the NAFLD group. The mean LSM was 5.3 (5.0,5.6) kpa in NAFLD group. Compared with the non-NAFLD group, the TyG, and its combination indicators with obesity (TyG-BMI and TyG-WC) and TG/HDL-c indexes were higher than the NAFLD group (all *p* < 0.001). See details for weighted characteristics of participants included in the study in Table 1.

**Table 1:** Weighted characteristics of participants included in the study

Items	Total (*n* = 1776)	Non-NAFLD (*n* = 992)	NAFLD (*n* = 784)	*p*-value
Age (years)	48.6 (46.9,50.3)	44.1 (42.3,46.0)	55.8 (53.9,57.7)	< 0.001
Gender = male (%)	48.8 (44.8,52.8)	42.8 (36.6,49.2)	58.3 (52.4,64.0)	< 0.001
Ethic = Non-Hispanic White (%)	63.1 (56.2,69.5)	65.2 (58.8,71.1)	59.9 (50.1,68.9)	0.114
Education = Less than high school (%)	10.4 (7.9,13.5)	8.9 (6.8,11.4)	12.9 (9.0,18.0)	0.008
Alcohol use (%)	90.7 (87.8,92.9)	91.0 (88.2,93.1)	90.2 (85.3,93.6)	0.681
Diabetes (%)	9.1 (7.9,10.5)	3.7 (2.6,5.3)	17.7 (14.8,21.0)	< 0.001
HTN (%)	24.2 (21.0,27.7)	16.5 (12.9,20.8)	36.5 (32.2,40.9)	< 0.001
Physical activity (%)	25.2 (20.9,30.1)	25.9 (20.3,32.3)	24.2 (19.3,29.9)	0.603
TG (mg/dL)	124.1 (118.3,129.9)	101.1 (97.1,105.1)	160.7 (151.1,170.4)	< 0.001
TC (mg/dL)	189.0 (185.4,192.6)	185.9 (181.3,190.6)	193.9 (189.4,198.4)	0.017
HDL-c (mg/dL)	55.8 (55.0,56.6)	58.7 (58.0,59.5)	51.1 (49.4,52.8)	< 0.001
BMI (kg/m^2^)	25.0 (24.7,25.2)	24.0 (23.7,24.3)	26.5 (26.1,26.9)	< 0.001
WC (cm)	89.8 (89.0,90.5)	85.9 (85.0,86.8)	95.9 (95.1,96.8)	< 0.001
HbA1c (%)	5.6 (5.5,5.6)	5.4 (5.4,5.4)	5.9 (5.8,5.9)	< 0.001
CAP (dB/m)	238.2 (234.3,242.1)	204.6 (201.5,207.8)	291.7 (287.7,295.6)	< 0.001
CAP categorical = NAFLD (%)	38.6 (33.9, 43.4)	0	100	< 0.001
TyG	8.5 (8.5,8.6)	8.3 (8.3,8.3)	8.8 (8.8,8.9)	< 0.001
TG/HDL	2.6 (2.5,2.8)	1.9 (1.8,2.0)	3.7 (3.4,4.1)	< 0.001
TyG-BMI	213.1 (210.4, 215.8)	199.8 (196.7, 203.0)	234.2 (230.2, 238.2)	< 0.001
TyG-WC	767.2 (758.2, 776.1)	716.0 (707.2, 724.8)	848.9 (837.4, 860.5)	< 0.001
LSM (Kpa)	4.9 (4.7, 5.0)	4.6 (4.4, 4.9)	5.3 (5.0, 5.6)	< 0.001
LSM categorical = liver stiffness (%)	6.4 (4.7, 8.7)	3.8 (2.8, 5.1)	10.6 (6.5, 16.6)	< 0.001

For continuous variables: survey-weighted mean (95% CI), *P*-value was by survey-weighted linear regression. For categorical variables: survey-weighted percentage (95% CI), *P*-value was by survey-weighted Chi-square test

*HTN* hypertension, *TG* Triglyceride, *TC* Total cholesterol, *HDL-c* High density lipoprotein cholesterol, *BMI* body mass index, *WC* waist circumference, *HbA1c* glycosylated hemoglobin, *CAP* Controlled attenuation parameter, *NAFLD* Non-alcoholic fatty liver disease, *TyG* Ln[TG (mg/dL) × FPG (mg/dL)/2], *TyG-BMI* TyG × BMI, *TyG-WC* TyG × WC, *TG/HDL-c* ratio of TG to HDL-c

Generalized linear regression models were performed to evaluate the relationship between CAP and NAFLD and the above indexes. After fully adjusting for age, BMI, gender, race, education level, alcohol use, diabetes, HTN, physical activity, and TC, TyG and its combination indexes TyG-BMI and TyG-WC were positively correlated with CAP with the βs (95%CI) of 24.810 (95%CI 21.339, 28.280) (*P* < 0.001), 0.704 (95% CI 0.597, 0.811) (*P* < 0.001) and 0.219 (95%CI 0.149, 0.289) (*P* < 0.001), respectively. TG/HDL-c also had a positive relationship with CAP [β = 2.983(95%CI 1.433, 4.533), *P* = 0.004]. See details in Table 2. After fully adjusting for age, BMI, gender, race, education level, alcohol use, diabetes, HTN, physical activity, and TC, a positive relationship between TyG, TyG-BMI, TyG-WC, and NAFLD was found with ORs of 3.387 (95%CI 2.328, 4.928) (*P* < 0.001), 1.032 (95% CI 1.019, 1.045) (*P* = 0.001) and 1.010 (95%CI 1.007, 1.013) (*P* = 0.002), respectively. TG/HDL-c also had a positive correlation with NAFLD with the β = 1.281 (95%CI 1.169, 1.403) (*P* = 0.006). See details in Table 2.

**Table 2:** The relationship between TyG, TyG-BMI, TyG-WC, TG/HDL-c and CAP and NAFLD (CAP ≥ 248 dB/m)

Exposure	CAP (dB/m) [β(95%CI) *p*-value]	NAFLD [OR (95%CI) *p*-value]
TyG	24.810 (21.339,28.280) < 0.001	3.387 (2.328, 4.928) < 0.001
TG/HDL	2.983 (1.433, 4.533) 0.004	1.281 (1.169, 1.403) 0.006
TyG-BMI	0.704 (0.597, 0.811) < 0.001	1.032 (1.019, 1.045) 0.001
TyG-WC	0.219 (0.149, 0.289) < 0.001	1.010 (1.007, 1.013) 0.002

Age, BMI, gender, ethic, education level, alcohol use, diabetes, HTN, physical activity and TC were adjusted. For the index of TyG-BMI, BMI was not adjusted

*TyG* Ln[TG (mg/dL) × FPG (mg/dL)/2], *TyG-BMI* TyG × BMI, *TyG-WC* TyG × WC, *TG/HDL-c* ratio of TG to HDL-c

Generalized linear regression models were used to assess the relationships between LSM and various indexes. After adjusting for age, BMI, gender, race, education level, alcohol use, diabetes, HTN, physical activity, and TC, we observed a positive association between TG/HDL-c and TyG-WC with LSM. The estimated βs (95%CI) were 0.057 (0.009, 0.105) and 0.004 (-0.000, 0.008) (*P* = 0.021 and 0.003), respectively. Additionally, TyG-WC showed a positive association with liver stiffness, with an OR (95% CI) of 1.006 (1.002, 1.012) (*P* = 0.013). Please refer to Table 3 for further details.

**Table 3:** The relationship between TyG, TyG-BMI, TyG-WC, TG/HDL-c and LSM and liver stiffness (LSM ≥ 7 kpa)

Exposure	LSM (kpa) [β(95%CI) *p*-value]	Liver stiffness [OR (95%CI) *p*-value]
TyG	0.382 (-0.115, 0.878) 0.071	1.555 (0.875, 2.763) 0.071
TG/HDL	0.057 (0.009, 0.105) 0.021	1.052 (0.998, 1.131) 0.112
TyG-BMI	0.004 (-0.004, 0.011) 0.234	1.004 (0.988, 1.020) 0.567
TyG-WC	0.004 (-0.000, 0.008) 0.003	1.006 (1.002, 1.012) 0.013

Age, BMI, gender, ethic, education level, alcohol use, diabetes, HTN, physical activity and TC were adjusted. For the index of TyG-BMI, BMI was not adjusted. TyG = Ln[TG (mg/dL) × FPG (mg/dL)/2]

*TyG-BMI* TyG × BMI, *TyG-WC* TyG × WC, *TG/HDL-c* ratio of TG to HDL-c

**Fig. 2:**
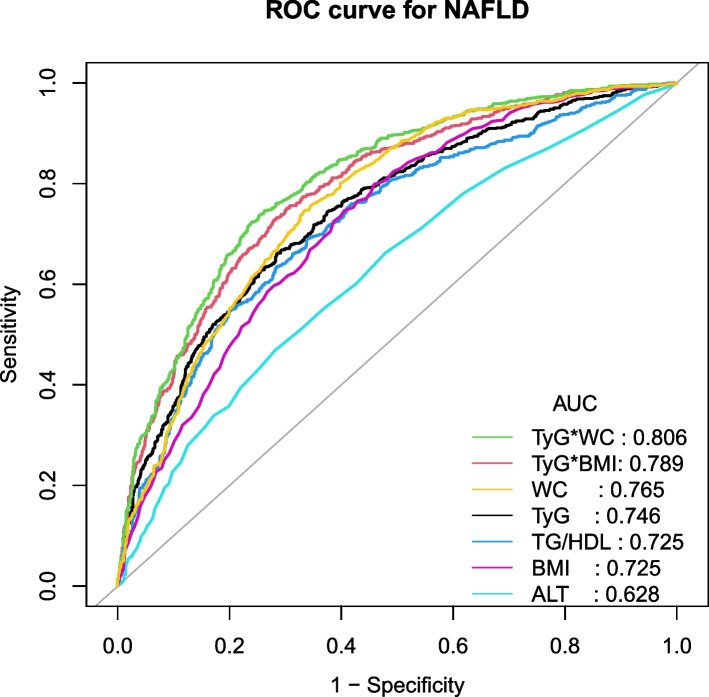
ROC curves for NAFLD

ROC curve analysis was performed to clarify the diagnostic capabilities of TyG, TyG-BMI, TyG-WC, TG/HDL-c, BMI, WC, and ALT for NAFLD in the non-obese population. Table 4 shows the diagnostic capability for NAFLD. In ascending order for AUC, the values of indexes were shown as followings: ALT [AUC = 0.628 (95%CI: 0.602, 0.654)], BMI [AUC = 0.725 (95%CI: 0.702, 0.748)], TG/HDL[AUC = 0.725 (95%CI: 0.701, 0.749)], TyG [AUC = 0.746 (95%CI: 0.723, 0.769)], WC[AUC = 0.765 (95%CI: 0.743, 0.787)], TyG-BMI [AUC = 0.789 (95%CI: 0.768, 0.810)] and TyG-WC [AUC = 0.806 (95%CI: 0.785, 0.826)]. TyG-WC index could be used to predict NAFLD with a specificity of 0.737, a sensitivity of 0.746, and the best threshold of 791.135. The ROC curves were shown in Fig. 2. TyG-WC was divided into 5 quantile groups (quantiles 1–5). The incidence of NAFLD in different quartile groups was 9.5, 18.4, 34.7, 58.5, and 83.1% (*P*-anova < 0.001). See details in Table 5. Supplementary Table 1 shows the diagnostic capability for liver stiffness. The TyG-WC were shown the highest value of AUC of 0.655(95%CI = 0.605, 0.706) compared with other indexes.

**Table 4:** AUC of TyG-BMI, TyG-WC, TyG, BMI, WC, ALT, and TG/HDL-C for diagnosing NAFLD

Variables	AUC	95%CI low	95%CI upp	Best threshold	Specificity	Sensitivity
TyG	0.746	0.723	0.769	8.598	0.714	0.662
TyG-BMI	0.789	0.768	0.810	219.810	0.698	0.751
TyG-WC	0.806	0.785	0.826	791.135	0.737	0.746
TG/HDL	0.725	0.701	0.749	2.184	0.716	0.635
ALT	0.628	0.602	0.654	19.500	0.718	0.470
BMI	0.725	0.702	0.748	25.050	0.595	0.746
WC	0.765	0.743	0.787	90.650	0.666	0.746

*TyG* Ln[TG (mg/dL) × FPG (mg/dL)/2], *TyG-BMI* TyG × BMI, *TyG-WC* TyG × WC, *TG/HDL-c* ration of TG to HDL-c, *BMI* body mass index, *WC* waist circumference, *ALT* Alanine aminotransferase, *AUC* area under curve

**Table 5:** The prevalence of NAFLD in 5 quantiles of TyG-WC

Groups for TyG-WC	Survey-weighted percentage for distribution (95%CI) (%)	Survey-weighted percentage for the prevalence of NAFLD (95%CI) (%)^a^
Quantiles 1 (476.1–661.4)	22.4 (19.3,25.8)	9.5 (5.1,16.9)
Quantiles 2 (661.4–747.1)	21.4 (18.8,24.2)	18.7 (15.0,23.1)
Quantiles 3 (747.1–812.9)	21.2 (17.9,24.9)	34.7 (25.5,45.3)
Quantiles 4 (812.9–884.4)	16.6 (14.5,19.0)	58.5 (51.1,65.5)
Quantiles 5 (884.4–1273.4)	18.4 (15.4,21.8)	83.1 (72.3,90.3)

^a^indicated *P*-anova < 0.001. *P*-anova for the prevalence of NAFLD in 5 quantiles groups was calculated by weighted linear regression model

## Discussion

Searching for a noninvasive and simple way to screen NAFLD has always been a hot topic for large-scale population studies. In this study, the relationship between NAFLD and the indexes of TyG, TyG-BMI, TyG-WC, and TG/HDL-c in the non-obese population was investigated; It was found that each of them was positively correlated with CAP and NAFLD. Among them, TyG-WC was the most effective in screening NAFLD in the non-obese population. This study also explored the relationship between liver cirrhosis and TyG and its combined indicators. Although TG/HDL-c and TyG-WC were positively correlated with LSM, only Tyg-WC and were independent risk factors for liver cirrhosis in non-obese population.

Due to different patient selections, diagnostic methods, BMI threshold, and lifestyles of the assessed population, the prevalence of non-obese NAFLD vary widely, ranging from 3 to 30% worldwide. A meta-analysis (2020) showed that around 40% of NAFLD occurs was non-obese population, and nearly 20% of NAFLD seemed to be lean individuals [16]. It emphasized that obesity should not be the only criterion for NAFLD screening. In this investigation, the prevalence of NAFLD, diagnosed by FibroScan, was 38.6% in the non-obese population, and it was 56.7% in the whole population diagnosed by the same cut-off value [17]. The estimated prevalence of non-alcoholic fatty liver disease (NAFLD) has been reported to vary widely, ranging from 7 to 26% [18]. It is worth noting that the prevalence of NAFLD in our study is relatively high. This could be attributed to the relatively low cutoff point used in the diagnostic criteria for NAFLD (CAP ≥ 248 db/m), as well as the relatively high cutoff point for non-obese diagnostic (BMI < 30 kg/m2). The high prevalence rate emphasizes the importance of identifying non-obese NAFLD through a simple and convenient method.

The pathophysiological mechanisms of non-obese NAFLD are similar to that of obese NAFLD. However, there are unique characteristics in the pathogenesis of non-obese NAFLD [19]. The key process is the overload of free fatty acids (FFAs), which makes the disposal mechanisms overwhelmed, thus leading to liver steatosis [20]. IR also contributed to NAFLD in both obese and non-obese populations, via increasing de novo lipogenesis in liver; Besides, IR can also indirectly promote the occurrence of NAFLD by reducing the inhibition of lipolysis in the fat depots and increasing the delivery of FFAs to the liver [21, 22]. It was reported visceral adiposity contributed as much as 50% to the level of FFAs in the portal vein [23]; Visceral fat accumulation in non-obese NAFLD was more serious than that in non-obese non-NAFLD; Therefore, visceral fat may be a promoting factor in the development of non-obese NAFLD. Since lean body mass is responsible for glucose disposal mediated by insulin, sarcopenia may cause impaired glucose tolerance and IR. In that case, sarcopenia was also reported to trigger non-obese NAFLD [19]. Genetic polymorphisms in *PNPLA3*, *TM6SF2*, *GCKR,* and *MBOAT7*, may also contribute to the occurrence of NAFLD in non-obese [24–26]. NAFLD in non-obese individuals could also develop into the decompensated end stage of cirrhosis [19]. Therefore, it is of great importance to identify NAFLD in non-obese individuals.

TyG, its combination with obesity and TG/HDL-c are novel indicators to evaluate IR in epidemiological studies. IR is closely related to NAFLD. TyG emerged as a new force, which was shown to be superior to HOMA-IR in evaluating IR [27–29]. A previous study demonstrated that the TyG index could be used to predict a diagnosis of NAFLD with AUC 0.763 in children [30]. TyG is also positively related to the development of liver fibrosis evaluated by fibrosis score and fibrosis-4 (Fib-4) in NAFLD patients [31]. In Chinese adults, a study found that TyG can effectively identify NAFLD evaluated by ultrasound scan, with AUC 0.782 and threshold 8.5 [32]. For TG/HDL-c, a study demonstrated that the TG/HDL-C ratio is associated with NAFLD with an AUC of 0.675 [33]. Waist circumference, as an index of visceral fat accumulation, is an important parameter to identify fatty liver in non-obese people. NAFLD patients had a greater WC than non-NAFLD in non-obese [34, 35]. Our study found that the combination of TyG and waist circumference (TyG-WC) was superior to other indicators in assessing NAFLD, which might be because the index reflects the combination of IR and visceral fat. This indicator is easily available and of great significance in screening NAFLD in a large-scale population study.

As far as we know, this is the first time to explore the relationship between liver steatosis (indicated by CAP) with TyG, TyG-BMI, TyG-WC, and TG / HDL-C in non-obese people. For every 1 unit increase in TyG, TyG-BMI, TyG-WC, and TG/HDL-c, the corresponding increases in CAP were 24.810, 2.983, 0.704, and 0.219 dB/m. It is observed that for non-obese NAFLD, lifestyle modification had little effect on reducing hepatic steatosis. However, this study reminds us that revising the above indexes may help to improve liver steatosis. Since the risk of NAFLD is closely associated with TyG-WC, interventions aiming to reduce triglyceride, glucose control, and abdominal obesity may be beneficial for decreasing the risk of NAFLD.

Liver biopsy remains the gold standard for diagnosis of NAFLD. However, due to the limitations of sampling variability, invasiveness, and high cost, liver biopsy cannot be used in large-scale epidemiological investigations [36]. Currently, ultrasound (US) B-mode imaging is the first-line method for diagnosing NAFLD, which allows subjective evaluation of fatty infiltration. However, it has low efficiency in detecting mild steatosis [37]. Magnetic resonance imaging-derived proton density fat fraction (MRI-PDFF) seems to be the most accurate noninvasive diagnostic method for NAFLD. However, MRI-PDFF cannot be widely used in the general population for high requirements for equipment. The controlled attenuation parameter (CAP) is a parameter based on ultrasonic signals, which is measured with M or XL probe by FibroScan ®. NAFLD could be diagnosed by CAP with an AUC of 0.82 at the cut-off value of 248 dB/m [15]. CAP represents the first-line method to screen NAFLD in the general population [38]. However, few studies have evaluated the accuracy of CAP in diagnosing NAFLD based on liver biopsy. More population-based studies are needed to clarify the optimal cut-off point for the diagnosis of NAFLD by CAP.

When exploring the relationship between LSM and TyG related indicators, we found that the diagnostic ability of TyG-WC in liver cirrhosis was not very prominent (AUC = 0.655) in non-obese individuals. This remains that TyG-WC was insufficient to predict liver fibrosis in the non-obese individuals. Previous studies have found that although TyG is associated with liver fibrosis in patients with NAFLD [31], it does not demonstrate sufficient diagnostic efficacy for liver fibrosis (AUC = 0.589) [39]. In obese and overweight without diabetes, the TyG-WC in diagnosis liver fibrosis was also relatively limited (AUC = 0.618) [40]. However, it was also found TyG index was independently associated with nonalcoholic steatohepatitis with AUC of 0.75 [41].

Cirrhosis has placed a significant economic and healthcare burden on numerous countries, particularly in the past three decades [14]. The influence of hepatitis B and C is projected to diminish in the near future, giving way to the prominence of non-alcoholic steatohepatitis (NASH) [42]. To achieve early detection of NAFLD, particularly in low-income countries, cost-effective interventions are essential. Fasting plasma glucose, triglycerides, and waist circumference are three easily obtainable physiological indicators. The TyG-WC, which incorporates these three indicators, can facilitate primary healthcare professionals in assessing participants' risk of NAFLD. The identification of this indicator holds valuable implications for the early diagnosis and treatment of NAFLD in non-obese individuals.

This study has several limitations. Firstly, the influence of medications used for glycemic control and dyslipidemia on TyG levels could not be accounted for in this research, which may introduce a confounding factor. Secondly, liver biopsy is considered the gold standard for diagnosing NAFLD, but it was not feasible due to the high risk and large sample size involved in this study. Therefore, alternative non-invasive methods were used to assess liver stiffness. Thirdly, the study participants were predominantly Americans, and caution should be exercised when extrapolating the findings to other ethnic populations worldwide. Different ethnic groups may have varying obesity standards, and this study did not conduct further subgroup analysis based on ethnicity.

## Conclusion

In the non-obese population, the indexes of TyG, TyG-BMI, TyG-WC, and TG/HDL-c were positively correlated with CAP and NAFLD. Among these indexes, TyG-WC was superior to others in identifying NAFLD.

### Supplementary Information

**Additional file 1: Supplementary Table 1.** AUC of TyG-BMI, TyG-WC, TyG, BMI, WC, ALT, and TG/HDL-C for diagnosing liver stiffness.

## Data Availability

The data that support the findings of this study are available from the corresponding author Dr. Mao upon reasonable request.
