# Supplementary material for: Triglyceride glucose-waist circumference: the optimum index to screen nonalcoholic fatty liver disease in non-obese adults
Source: BMC Gastroenterol. 2023 Nov 2;23:376. doi: 10.1186/s12876-023-03007-8 (PMC10621119; doi:10.1186/s12876-023-03007-8)
Supplement: Supplementary file 1 — Additional file 1: Supplementary Table 1. AUC of TyG-BMI, TyG-WC, TyG, BMI, WC, ALT, and TG/HDL-C for diagnosing liver stiffness. [file 12876_2023_3007_MOESM1_ESM.docx]

Supplementary Table 1: AUC of TyG-BMI, TyG-WC, TyG, BMI, WC, ALT, and TG/HDL-C for diagnosing liver stiffness.

| Variables | AUC | 95%CI low | 95%CI upp | Best threshold | Specificity | Sensitivity |
| --- | --- | --- | --- | --- | --- | --- |
| TyG-WC | 0.655 | 0.605 | 0.706 | 808.963 | 0.608 | 0.676 |
| TG/HDL | 0.608 | 0.559 | 0.657 | 2.440 | 0.624 | 0.566 |
| ALT | 0.588 | 0.537 | 0.639 | 17.500 | 0.563 | 0.580 |
| BMI | 0.575 | 0.523 | 0.627 | 27.250 | 0.706 | 0.462 |
| WC | 0.641 | 0.591 | 0.690 | 94.950 | 0.663 | 0.568 |
